# Men’s impulsivity underpins gender differences in aggressive behaviour

**DOI:** 10.1038/s41598-025-20114-6

**Published:** 2025-10-09

**Authors:** Annah G. McCurry, David I. Donaldson, Robert C. May

**Affiliations:** School of Psychology and Neuroscience, The University of St Andrews, St Mary’s Quad, South St, St Andrews, KY16 9JP UK

**Keywords:** Psychology, Human behaviour

## Abstract

The relationship between gender and aggression is multi-faceted, with survey research showing that the aggression varies with the gender of both aggressor and target. Here, using a lab-based paradigm that controls the capacity to cause harm and the risk of retaliation, we reveal pronounced gender-specific differences in aggression.Reactive aggression was assessed using a competitive reaction-time task that allowed dyadic pairs to interact face-to-face during conflict. Across two studies (*n* = 162/*trials* = 5099) same- and mixed-gender familiar pairs blasted each other repeatedly with noxious sounds, producing an oscillatory pattern of escalating and de-escalating aggression. Analysis revealed lower aggression in dyads made up of two women than mixed dyads or those made up of two men. Crucially, men were an order of magnitude more likely than women to initiate increases in aggression, and brief experimentally imposed delays designed to block impulsive behaviour significantly reduced male aggression. Put simply, when higher levels of aggression were seen, men (usually) started it.

**Supplementary Information:**

The online version contains supplementary material available at 10.1038/s41598-025-20114-6.

## Introduction

The role of gender in aggression is a contentious topic and researchers examining differences in the propensity of men and women to engage in aggression have reported mixed results^1–5^. Some of the mixed results could be explained by gender differences in risk-taking and impulsivity, both of which are central concepts in theories explaining aggressive behaviour. Empirical evidence suggests that men are more likely than women to engage in behaviour that is risky, behaviour that is impulsive, and behaviour that is both risky and impulsive^2,3^, which could account for observed gender differences in aggression in both lab-based studies and real-world statistics. However, teasing apart the influence of gender, risk-taking, and impulsivity on aggression is difficult in practice because the risks of a potentially aggressive encounter are impacted by both the gender of the could-be aggressor and the could-be target of aggression^6–8^. Consequently, in the present article we aim to untangle these complex factors, examining the role of (both aggressor and target) gender on aggression while controlling for variability in risk and manipulating the effects of impulsivity.

### Theoretical considerations

The term “aggression” encompasses a wide array of behaviours that are intended to harm another person^9,10^. This harm can take a variety of forms, including physical (i.e., injury from a blow), verbal (i.e., upset caused by name calling) and social (i.e., someone spreading harmful rumours about you to your peers). Some types of aggression can be hard to separate in practice, due to overlap in the mechanism or type of harm caused (e.g., verbal vs. emotional), and because different types of aggression often co-occur (e.g., name calling and threatening before or during a physical altercation). Nonetheless, studies reveal that gender differences in aggression vary with the form and function of aggression^1,2^, making it particularly important to distinguish between physical and non-physical forms of aggression. Studies typically show that men are much more likely than women to engage in physical forms of aggression^1,6,11^. When women engage in aggression, they typically use non-physical, less risky forms.

Before discussing risk-taking and impulsivity in depth, we should clarify the difference between “reactive” and “proactive” aggression. By definition, reactive aggression refers to aggression in response to a perceived threat or provocation^12^.It is therefore believed to be immediately preceded by some negative affect and enacted impulsively^2,12^. By contrast, proactive aggression is seen as relatively unemotional, goal oriented, and deliberate. Most commonly, studies of human aggression—including the present article—are concerned primarily with reactive forms of aggression. Below we describe how our paradigm elicits reactive aggression, but for brevity, we will use “aggression” to refer to “reactive aggression” unless otherwise indicated.

Risk-taking and impulsivity are important factors in theories of (reactive) aggression. For example, the General Aggression Model (GAM)^9,13^ argues that the difference between impulsive and “thoughtful” action is the difference between aggressive and non-aggressive behaviour. In other words, the GAM suggests that impulsivity leads to aggression, and that factors like negative affect and provocation increase the likelihood of impulsivity and aggression. Similarly, the I^3^ Model^14–19^ implicates “inhibition” (the inverse of impulsivity) as the linchpin for avoiding aggressive behaviour in the presence of negative affect (“impellance”) and provocation (“instigation”). While there is empirical evidence supporting gender differences in risk-taking and impulsivity^2–5,16–22^, some of which appear in infancy^23^, the biosocial and evolutionary theories that attempt to explain these observed sex-differences are outside the scope of the present article. Whatever the reason, women are, on average, less likely than men to take risks and engage in impulsive behaviour.

The relationship between gender and aggression is difficult to untangle because the gender of the target of aggression, not just the gender of the aggressor, is likely to impact aggressive behaviour. This causes particular difficulties in research into heterosexual intimate partner aggression, where the gender of the aggressor and target are effectively confounded. Importantly, experimental research has the potential to investigate gender differences in aggression by limiting and equalizing the capacity of men and women to cause harm (while controlling for variability in risk as a factor of target-gender). To date, however, the majority of experimental research examining gender effects on aggression use only one participant^e.g.,^^24,25^ (rather than dyads participating together), making it impossible to examine the impact of both aggressor and target gender. Below, we outline our new face-to-face, dyadic methodological approach that allows us to hold the risk associated with physical aggression constant for both men and women (regardless of target gender), whilst manipulating the opportunity for participants to act impulsively, and examining dyadic gender interactions in reactive aggression.

### Methodological constraints and innovations

To date, the majority of studies examining gender differences in aggression have employed non-dyadic (i.e., single-participant) designs that do not allow for the investigation of both aggressor and target gender effects. For example, classic lab-based aggression tasks (e.g., the Competitive Reaction Time Task/Taylor Aggression Paradigm; CRTT/TAP) typically manufacture artificial conflict between a participant and a fake opponent (a computer program)^26,27^. Alternative hypothetical scenario methods (e.g., Articulated Thoughts in Simulated Situations; ATSS) do not rely on deception and can include target-gender manipulations, but they require participants to introspect about how aggressive they think they would be in imagined situations (rather than measuring aggression behaviourally)^6,28^. Further, existing dyadic studies that examine aggressor-target gender interactions are limited insofar as they are not commonly experimental; they usually employ online^29,30^ or retrospective^31^ self-reports of aggression, where gender differences in reporting behaviour confounds results^32^. Equally, the uncontrolled nature of real conflict makes it extremely difficult to examine the causes of differences in men’s and women’s aggression based on observations ‘in the wild’. As noted above, for example, conflict behaviour is affected both by differences in the capacity of men and women to cause physical harm^20^, and variability in the risk of retaliation based on context (i.e., in public or behind closed doors^33–35^).

In response to these challenges, the current study investigates gender differences in aggressive behaviour in a lab setting, examining the influence of aggressor-target gender on aggression whilst holding constant both the risk of retaliation and the capacity of each individual to cause harm. We had familiar dyads (couples or friends, combining data across two studies) interact with one another, face-to-face, in a competitive reaction time task (the FTF-CRTT^36^; see the Methods section for more details). Critically, the FTF-CRTT defines upper and lower limits of aggression as sound blasts (short blasts of noxious noise delivered via headphones) where the capacity to cause harm/pain, therefore, is the same for all participants regardless of aggressor and target gender. This is important because it ensures that while the amount of aggression can vary over time, the threat of some degree of retaliation from every opponent is certain (i.e., the risk is always greater than zero) and the capacity of men and women to cause harm is equal (i.e., everyone uses the same 1–8 scale). In sum, therefore, the FTF-CRTT is not only innovative because it involves live in-person interaction between individuals, but also because it allows us to hold both the risk of retaliation and the scale of that risk constant for all gender pairs.

Our FTF-CRTT task expands on previous studies that had two real participants compete virtually^e.g.,^^26,37^. Crucially, however, our approach allows for the careful examination of complex interpersonal processes^38^, including time-lagged analysis^39^ of the impact of past behaviour from both participants on subsequent aggression (during the task), revealing the on-going dynamics of conflict. Several previous studies have attempted to use non-FTF versions of the CRTT/TAP for gender-effect investigations. For example, Koch et al. (2024)^37^ had same-gender sibling pairs engage virtually in a rock-paper-scissors variation of the TAP, where the winner of each round was allowed to subtract between 0 and 100 cents from their opponent, finding no significant differences between male-male and female-female sibling pairs^37^. However, other TAP and TAP-variant studies have shown significant effects of gender, particularly when examining reactive aggression in both its physical^24^ and non-physical^40,41^ forms (though these studies have only used one real participant and a fake opponent, so they have not been able to examine aggressor-target gender effects).

Importantly, the current studies build on our previous publication using the FTF-CRTT^36^ by focusing here on gender dynamics, including both same- and mixed-gender pairs, allowing us to examine variation in aggressive behaviour associated with both aggressor and target gender. In addition, because impulsivity is a hypothesised mechanism underlying gender differences, we also implemented an experimental manipulation designed to prevent impulsive behaviour during a provoked state (i.e., blocking negative urgency). Specifically, we introduced “forced breaks”, brief delays between provocation and the opportunity for aggression that are known to reduce aggression by blocking impulsive behaviour^36^. If higher aggression in men than women is indeed underpinned by a sex difference in impulsivity, we should expect these forced breaks to reduce men’s aggression to a greater extent than women’s aggression.

Four specific research questions provide a structure for our reporting of the data. First, we ask if men and women differ in aggression in the FTF-CRTT. To answer this question, we compare mean aggression in men and women globally, as well as in men-only pairs, women-only pairs, and mixed-gender pairs. Critically, within mixed-gender pairs, we assess whether women are equally as aggressive with other women as they are with men. Second, we ask if aggressive conflict follows a predictable pattern (i.e., what is the pattern of aggression over time?), and if so, does this pattern differ by the gender of the aggressor and target? To answer this question, we employ a statistical modelling approach that allows us to examine the impact of each person’s behaviour (and their opponent’s behaviour) from one round to the next. Specifically, we use a coupled linear oscillator model, which captures information about both participants’ aggression trajectory over time, allowing us to explore the complex interactions that are present in dyadic data (full details of the coupled linear oscillator model are provided in the methods and results sections, and in the Appendix).

Having characterised the overall pattern of aggression, next we turn to issues raised by the theoretical accounts of aggression outlined above. As such, our third research question concerns accounts of the role of impulsivity in mediating men’s aggression. Specifically, we ask if forced breaks that are designed to prevent risky impulsive behaviour have a larger aggression-reduction effect in men than women. Finally, our fourth question examines the theoretical expectation that women prefer non-physical forms of aggression. We test whether women used spontaneous verbal aggression *instead of* physical aggression in the present study, using a post-hoc analysis of verbal aggression. Our task was not designed to assess verbal aggression, but we were able to address this issue because we have audio recordings of each experimental session. We, therefore, present an analysis of dyad-level patterns of verbal aggression in the results.

## Methods

### Study 1: couples

#### Participants

A total of 104 participants (reported previously in McCurry et. al., 2024; 60 women, 3 non-binary participants; assessed using the item “what is your gender?” in the post-experiment questionnaire) were recruited from a participant pool at the University of St Andrews in Scotland. Because the analyses presented in this paper use gender as a categorical predictor, we use a subset of our original dataset, only including participants who identified as either a man or a woman and who agreed that their gender is aligned with their sex assigned at birth. The full dataset is available online and analyses of the full dataset are available in McCurry et al., 2024^36^. Participants were romantic couples (of any orientation) who participated together, and each partner was given £12.50 in compensation. Participants’ mean age was 21.1 years (*SD* = 3.3) with a minimum of 18 and maximum of 39. Most participants were full-time students (72.8%) and reported their ethnicity as White (70.3%). Most couples were not cohabiting (66.1%), and the average duration of a relationship was roughly eight months. This study was approved by the School of Psychology and Neuroscience Ethics Committee in December 2022 (approval code PS16636) before data collection commenced (and all methods were performed in accordance with the approved ethics application and all other relevant guidelines and regulations). Informed consent from participants was obtained in person with physical consent forms; procedure described below.

#### Design

We used a between-groups design. Dyads were recruited either as romantic couples or friend pairs and assigned randomly upon arrival (and without their knowledge) either to the Immediate Response (*n* = 23) or one of the Forced Break conditions (5 s: *n* = 32; 10 s: *n* = 23; and 15 s: *n* = 26; total *n* = 81). The study was not pre-registered.

#### Materials

We assessed trait aggression using the short form of the Buss and Perry Aggression Questionnaire (BPAQ^42^; reported in our previous paper^36^) and we measured aggressive behaviour with a modified version of the Competitive Reaction Time Task (CRTT)^43^ where our participants competed against each other, face-to-face (the FTF-CRTT)^36^. Our Face-To-Face CRTT (FTF-CRTT) was minimally rigged: when participants’ reaction times were within 100 ms of each other, a winner was selected at random. When one participant’s reaction time was more than 100 ms later than their partner, the faster participant won the round. This was designed to avoid suspicion of rigging (pilot testing showed that participants could auditorily distinguish whose button press reaction time was faster when there was more than a 100 ms gap) while keeping the win-loss rate of each participant close to 0.5 (so that all players had roughly equal opportunity to aggress). Higher scores (i.e., where the winner chooses a louder blast to play to the loser of the round) are indicative of more aggression. Materials were identical in both samples; further details are available in our previous paper^36^ (which focused instead on the effects of emotion, and only included data from couples). Finally, we recorded video and audio data using an Insta360 camera placed in-between participants below face height (so dyads could see each other unobstructed). Video specifications are not provided here as we do not report this data in the present article, but interested readers can see McCurry et al. (2024)^36^ for details on video recording.

#### Procedures

The study was advertised using University of St Andrews’ internal participant recruitment and memo systems as a study of competitive reaction time (omitting our focus on aggression). Upon arrival to the lab, participants read information sheets and signed consent forms. After being given an opportunity to ask questions, participants were shown a demonstration of the game, where they were able to hear the lowest, medium, and highest blast levels (1, 4, and 8, played at 75db, 90db, and 110db respectively). Participants were then prompted to put on their headphones and stand in front of their monitors. Each participant had a set of headphones, a large arcade button, and a keyboard. The participant stations were set up such that participants stood facing each other, with no visual obstructions. Further details are available in McCurry et al. (2024)^36^.

Once participants were ready to begin, our custom FTF-CRTT interface (coded in Python^44^) appeared on participants’ screens. They were prompted to enter their names and begin the game. Each round started with a black screen displaying “Ready” in white text. One second later, “Set” appeared, and then 0–8 s later (time length was randomized to increase task attention), “GO!!” appeared, signalling the participants to hit their arcade buttons as quickly as possible. The names of the winner and loser of the round were displayed. The winner was then prompted to enter their selected blast level on their keyboard and the blast was sent to the loser’s headphones. The next round would begin immediately once the blast ended, and the game continued for 30 rounds. In the control “Immediate Response” condition (which functions as our baseline), participants were able to select the blast level straight away, whereas in the experimental “Forced Break” conditions, participants were unable to select a blast level for 5, 10, or 15 s after the winner was announced. We used a between-subject design and multiple forced break lengths to assess whether the impact of forced breaks varied by their length. Because the length of forced breaks did not affect results^36^, these are collapsed into a single Forced Break condition.

Once all 30 rounds were complete, participants filled in a post-experimental questionnaire containing the BPAQ-SF and custom measures of self- and other- emotion and aggression. We also prompted participants to (optionally) indicate how they felt about their experience overall in a free text answer box as part of the debrief. Participants were then debriefed and told of the true intentions of the study (to examine aggression).

### Study 2: friends

#### Participants

A total of 58 participants (46 women) were recruited from a participant pool at the University of St Andrews in Scotland. Participants were friends who participated together, and each participant was given £10 in compensation. Participants’ mean age was 24.6 years (*SD* = 3.7) with a minimum of 17 and maximum of 36. Most participants were full-time students (90.0%) and Asian (79%). Most participants were not cohabiting (71%), and the average duration of the friendship was roughly ten months. This study was approved by the School of Psychology and Neuroscience Ethics Committee (as an amendment) in January 2024 (approval code PS16636) before data collection commenced (and all methods were performed in accordance with the approved ethics application and all other relevant guidelines and regulations). Informed consent from participants was obtained in person with physical consent forms; procedure described below.

#### Design

We used a between-groups design. Dyads were assigned randomly upon arrival (and without their knowledge) either to the ten second Forced Break condition (*n* = 18) or one of the Immediate Response conditions (Immediate Response: *n* = 18; and three-second time-out/Forced Response: *n* = 22). In the time-out condition, participants are forced to select a blast within three seconds of the winner being announced. If they fail to respond within three seconds, they receive a blast (randomly selected between four and eight) and then the game continues onto the next round. The study was not pre-registered.

#### Materials

All materials used in the primary study were used in the follow-on study. We included several additional self-report measures (the Difficulties in Emotion Regulation Scale^45^ and the Negative Urgency subscale of the Urgency-Premeditation-Perseverance-Sensation Seeking scale^46^) that are not reported here.

#### Procedures

Procedure in the second study was identical to the first study, with minor modifications to the conditions. Specifically, the friend study used only one Forced Break condition with breaks of 10 s. Further, we introduced a second kind of quick response condition. The new kind of quick response condition is a “Forced Response” condition wherein participants must select a blast level within three-seconds of the winner being announced (and if they fail to respond, the program blasts the winner with a random blast selection between four and eight).

### Statistical analysis: both studies

In the present analysis (and in line with our previous reporting^36^), we collapse all Forced Break conditions for simplicity because data from the Forced Break conditions do not differ significantly from one another. Similarly, we collapse our Immediate Response and our Forced Response conditions for the same reasons (i.e., simplicity and lack of differentiation between the standard Immediate Response and the new time-out condition).

*Null Hypothesis Significant Testing Statistics.* All analyses were performed in R using two-tailed analysis. All regressions were performed using the Linear Least Squares method. All other comparison of means testing used Wilcoxon Rank Sum tests with a Continuity Correction because Shapiro–Wilk Tests for Normality revealed that data was significantly non-normal (see Appendix A; note Wilcoxon are better able to accommodate normal data than t-tests); equal variance was assumed following a visual inspection of the data. The effect size, r, was calculated from the Wilcoxon tests using the wilcox_effsize function of the rstatix package (version 0.7.2). Full statistical information is available in Appendix A. Where possible, analysis was conducted with trial-level data to extract as much information from the data recorded as possible and avoid biasing results with artificially normal (averaged) data with deceptively low variance^47^. Finally, full models are presented in the Appendix (B-E). Normality of residuals for all models was assumed.

*Bayesian Statistics.* All Bayesian analysis^48,49^ was performed in R using the *BayesFactor* package (version 0.9.12–4.6. For comparison of means testing, the *ttestBF* function was used with Jeffreys prior applied to the variance of the normal prior sample (i.e., a minimally informative prior) and a Cauchy prior applied to the effect estimate^50,51^. For correlation analysis, the *regressionBF* function was used with the same arrangement of priors. All grey shaded regions shown in plots represent standard errors. All boxplot elements represent their defaults in ggplot2 (i.e., centre line, median; upper and lower box boundaries, Inter Quartile Range (IQR); upper and lower whiskers, smallest or largest value within 1.5 × the corresponding IQR; dots, outliers). We use a combination of Bayesian and NHST statistics for added rigour and to support engagement from the widest possible audience.

*Nested, Multi-Level, Cross-Lagged, Coupled Linear Oscillator Modelling.* Our Coupled Linear Oscillator models were inspired by Hilpert 2020^38^ and produced in R using linear least squares modelling. To use our data efficiently, we employed cross-lagged analysis to calculate how different factors influence each other over time. We further used a nested multilevel design to account for variation caused by individuals and dyads. Full model details are available in Supplemental Information.

*Imbalanced Data Corrections.* As indicated by the participant demographics from both studies, we have imbalance in a.) the number of men and women who participated (total 106 women and 53 men) and b.) the distribution of same- vs. opposite- gender pairs across our two samples (i.e., study two had more same-gender pairs). To address gender-based sample size unbalances (a) in statistical tests, we only used tests that are insensitive to (or mathematically account for) differences in between-group differences in sample size and or variance, such as the Wilcoxon Rank Sum test^52^ when imbalance is present. Further, we note that our use of a Bayesian equivalent to a t-test is also appropriate given unbalanced data^53^. We also wish to note that our data used in our logistic regression is balanced as it uses only mixed-gender pairs. Finally, to address imbalances in the distribution of same- and opposite gender pairs across our two studies (b), we conducted an analysis of the impact of study (i.e., study one or two) and gender presented in Appendix D. There is evidence that sample origin (couples vs friends) affected blast level selections, but there is about 23 times as much evidence that participant gender has an effect (i.e., the evidence that aggression varies by gender is much stronger than the evidence that it varies by sample).

## Results

### Are men more aggressive than women?

**Fig. 1:**
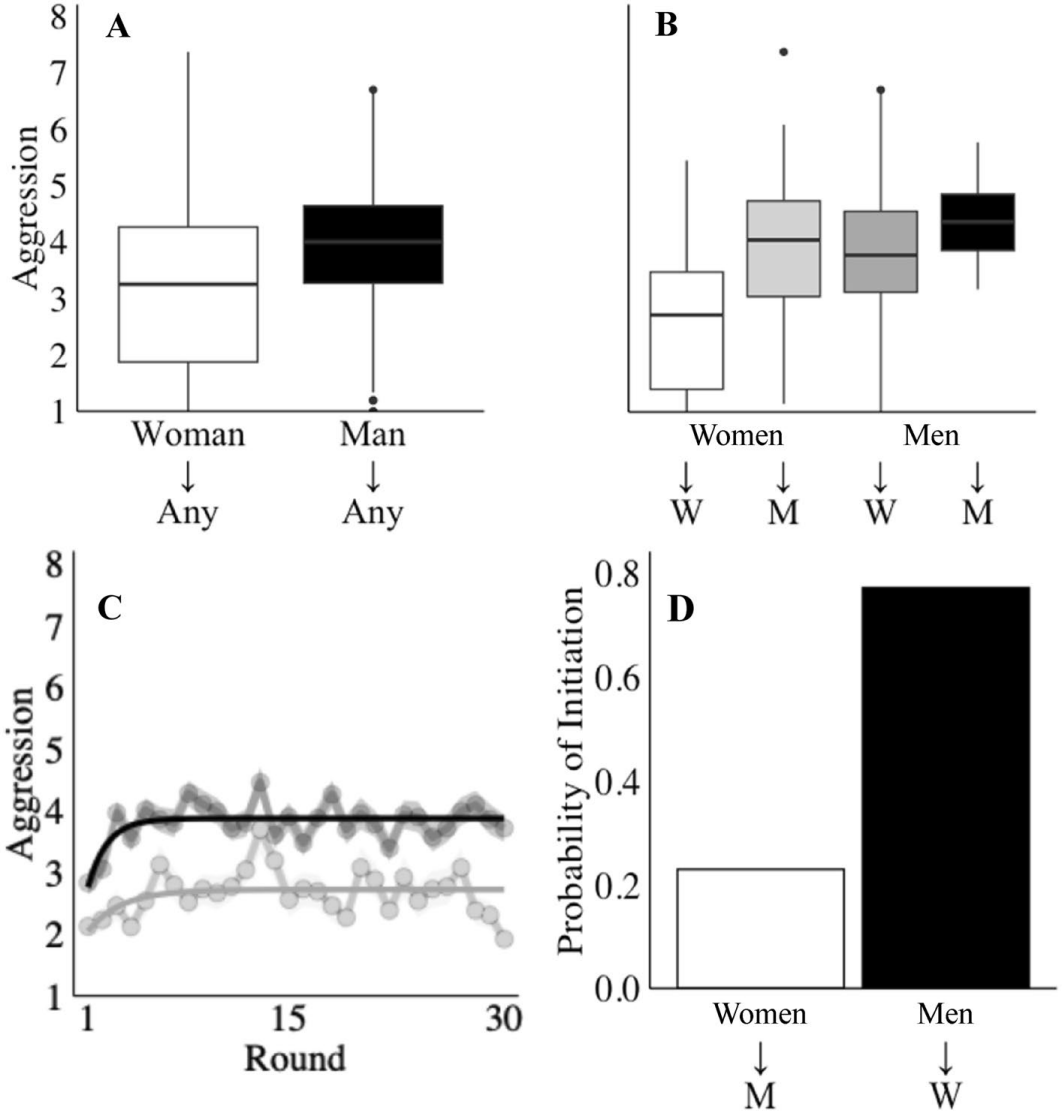
Gender differences in aggression. (**A**) Behaviour, averaged across all rounds and gender pair combinations, reveals that overall mean aggression levels were higher for men than women. (**B**) Overall differences in aggression between men and women reflect an interaction between aggressor and target gender. Specifically, all pairs containing at least one man behaved similarly, but women-only pairs were less aggressive (and these pairings pull down the overall average for women). (**C**) Aggression over time, averaged by round, shows that women only pairs (white) consistently exhibit less aggression than pairs containing at least one man (dark grey). (**D**) The probability of men and women initiating an escalation in aggression, averaged across mixed gender pairs, reveals a large gender difference in who is most likely to initiate an increase in aggression. Specifically, men are an order of magnitude more likely than women to raise aggression above the pair’s overall mean.

As expected, and confirming our first hypothesis, analysis of behaviour averaged across all rounds and gender pair combinations shows that men were significantly more aggressive than women overall (Fig. 1A; *p* = 0.006, r = 0.219, bf = 7.767; all statistics for all results are available in Table 1 and additional information is available in the Extended Data). The overall effect of gender is driven, however, by the fact that pairs containing two women were significantly less aggressive than pairs containing at least one man (Fig. 1B; *p* = 1e-7, r = 0.421, bf = 300,088). Counter to our second hypothesis, therefore, women consistently engaged in more aggression against men than against other woman (*p* = 6e-6, r = 0.451, bf = 5949). Reinforcing this finding, examination of the data over successive rounds shows that women-only pairs consistently exhibited less aggression than pairs containing at least one man (Fig. 1C; *p* = 2e-10, r = 0.707, bf = 7e+14). Given our control over the threat of retaliation (where some degree of retaliation is certain) and the capacity for harm (which is equal for both genders), these findings clearly warrant further investigation.

**Table 1:** Full statistics.

Sample	Test	Panel	n	k	Effect Size	*P*-value	Bayes Factor	95% CI ↓	95% CI ↑	df	Stat
Figure 1											
Winning Players	Wilcoxon Signed-Rank Test	A	160	NA	0.219	**0.006**	7.767	0.200	1.250	158	3714.5
		B (WW, WM)	101	NA	0.451	**6e-6**	5949	− 1.920	− 0.773	99	607.5
		B (WW, MW)	100	NA	0.412	**4e-5**	1705	− 1.854	− 0.705	98	651.5
		B (WW, MM)	59	NA	0.453	**0.001**	113	− 2.816	− 0.921	57	47
		B (WM, MW)	99	NA	0.029	0.774	0.218	− 0.464	0.650	97	1266.5
		B (WM, MM)	58	NA	0.115	0.385	0.516	− 1.406	0.420	56	161
		B (MW, MM)	57	NA	0.131	0.329	0.565	− 1.450	0.378	55	153
		C	60	NA	0.707	**2e-10**	7e-14	− 1.342	− 1.002	58	18
Winning Players	Logistic Regression	D	96	NA	2.426	**6e-7**	4e + 6	1.510	3.424	97	4.995
Figure 6											
Winning Players in a Forced Break Condition	Wilcoxon Signed-Rank Test	A (WW)	51	1540	0.039	0.117	0.735	− 3.633	2.948	1538	277,348
		A (M +)	104	3213	0.116	**5e-11**	1e + 7	2e-5	0.999	3211	1e +
		A (WW, M +)	155	NA	0.314	**9e-5**	402	2e-5	0.999	153	1655
All Players	Wilcoxon Signed-Rank Test	B (WW, M +)	103	NA	0.336	**6e-4**	18.3	− 3e-5	− 2.999	101	825

Significant values are in bold.

To discover why pairs with two women were less aggressive, we first examined levels of aggression by gender of aggressor within mixed-gender pairs, asking whether men and women behaved differently when interacting with each other. This focused analysis revealed no evidence of a difference in the level of aggression exhibited by men and women within mixed-gender pairs (cf. Figure 1B; *p* = 0.774, r = 0.029, bf = 0.218). In addition, we also examined the pattern of mixed gender aggression over time, assessing which member of each mixed-gender pair was responsible for the *initiation* of heightened aggression (defined as the first instance of substantive escalation, i.e., a blast at or above the pair’s overall mean). Crucially, analysis revealed that the initiation of heightened aggression was 11 times more likely to come from a man than a woman (Fig. 1D; *p* = 6e-7, r = 0.223, bf = 4,192,080). This finding shows that, on average, it was men that initiated the aggressive cycle in mixed-gender pairs, with women then raising their aggression level to match. Given the significant difference in the way men and women initiated heightened conflict, we conducted further analysis to assess gender differences in other aspects of conflict escalation and retaliation.

### Does aggression follow a predictable pattern?

**Fig. 2:**
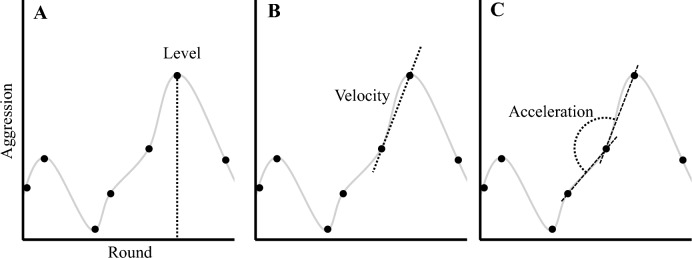
Metrics for modelling conflict behaviour. When accounting for an aggressor’s next blast level, we are interested in their previous behaviour, as well as the target’s previous behaviour. We introduce three variables to model previous behaviour in each member of a pair: (**A**) Level refers to aggression (i.e., blast selection) in the most recent round; (**B**) Velocity is the change in aggression (i.e., level) across the two most recent rounds; and (**C**) Acceleration is the change in velocity across the three most recent rounds. We examined each pairs behaviour, over all successive rounds, using a Cross-Lagged Coupled Linear Oscillator Model to produce a stationary account that shows the relationship between current behaviour and each individual’s previous aggression (i.e., level), trajectory (i.e., velocity), and volitivity (i.e., acceleration).

To characterise the patterns of aggression expressed during our task we examined behaviour using a Cross-Lagged Coupled Linear Oscillator Model^38,39,54–56^ (inspired in particular by Hilpert, 2020)^38^. This approach allows data from the entire interaction between each dyadic pair to be incorporated, capturing the dynamics of conflict over successive rounds of the task. Within the model “level” refers to the blast level chosen on a given round (Fig. 2A); “velocity” is the change from one round to the next and thus represents round-to-round escalation (Fig. 2B); finally, “acceleration” is the change in velocity, such that higher (positive) acceleration indicates an increase in the rate of escalation (Fig. 2C). Modelling the data in this way allows us to show how an aggressor’s behaviour on a given round is influenced by behaviour from previous rounds (including their target’s prior behaviour). Using this approach we first present a general model, describing overall aggression trends (i.e., ignoring gender and including all aggressor and target combinations), measured across all pairs. For completeness we provide *p* values, unstandardized and standardised betas, and a Bayes Factor for each model (taken from a separate Bayesian analysis, see supplementary information in the Extended Data). Note that in the context of Bayesian regression modeling, the Bayes Factors reflect the evidence for including versus excluding a given variable in a model (not the strength of evidence for or against a hypothesis) by providing the relative evidence for a given model with the variable of interest over the same model without that variable. Bayes Factors reported here, therefore, facilitate model comparison by quantifying how much more likely the data are under one model specification than another.

**Fig. 3:**
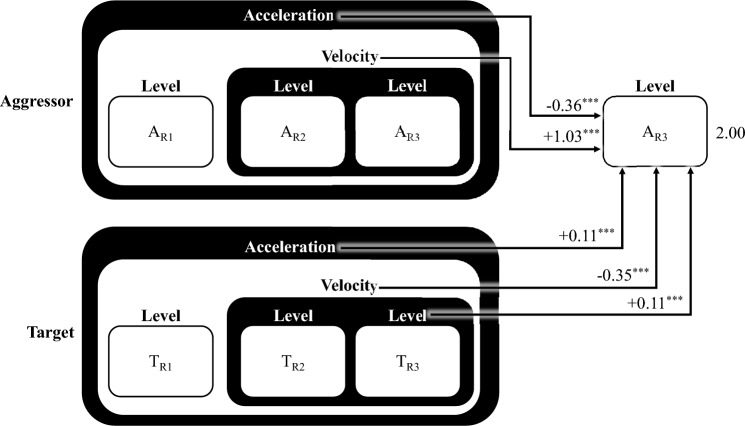
General cross-lagged, coupled oscillatory model of aggression. A general model representing overall aggression trends based on data from all pairs. The model attempts to account for the aggressor’s next blast level selection (A_R3_) based on their preceding level, velocity and acceleration, and their target’s preceding level, velocity, and acceleration. The number to the right of the aggressor’s next blast level is the intercept of the model (2.00), indicating the predicted blast level if all input variables are zero. The number along the paths from each predictor to the aggressor’s next blast level represent the increase in predicted blast level for every one unit increase in the associated predictor. For example, if the aggressor’s velocity increases one unit, we expect a 1.03 unit increase in their subsequent blast. This means that if the aggressor’s velocity is 1, and all else in the model is zero, the predicted blast is 3.03 (i.e., 2.00 + 1.03). All predictors are strongly significant, and the general model explains ~ 74% of variance in the aggressor’s next blast level selection.

The general model accounts for ~ 74% of variance in the aggressor’s blast level selection, with all modelled factors being strongly significant (*p* =  < 0.001, Marginal R^2^ = 0.514, Conditional R^2^ = 0.743). As shown in Fig. 3, the general model reveals the influence of the target’s previous blast level on the aggressor’s current blast level, reflecting the fact that participants tend to match each other (*p* =  < 0.001, B = 0.44, β = 0.43, bf = 2e + 7). The model also shows that an aggressor’s own increased velocity (escalation from round-to-round) predicts higher blast selections (*p* =  < 0.001, B = 1.03, β = 0.97, bf = 8e + 27), whereas their acceleration (change in the rate of escalation) predicts a subsequent decrease in blast levels (*p* =  < 0.001, B = -0.36, β = -0.59, bf = 2e + 12). That is, increases in aggression (i.e., velocity) are initially associated with further increases (i.e., escalation), whereas continued increases over time (i.e., acceleration) are associated with decreases in aggression (i.e., de-escalation). Interestingly, we see the opposite direction of effect in relation to the target’s velocity (*p* =  < 0.001, B = -0.35, β = -0.03, bf = 305) and acceleration (*p* =  < 0.001, B = 0.11, β = 0.20, bf = 0.180), such that earlier increases in velocity by the target lead to reduced blast level selections by the aggressor, whereas earlier increases in acceleration lead to increases in aggression (although note that in the latter case the Bayes Factor is anecdotal, indicating that the Bayesian versions of our model with and without this factor performed similarly). Full model specifications, including a standardized version of the same model, and a Bayesian equivalent are provided in Appendix B.

**Fig. 4:**
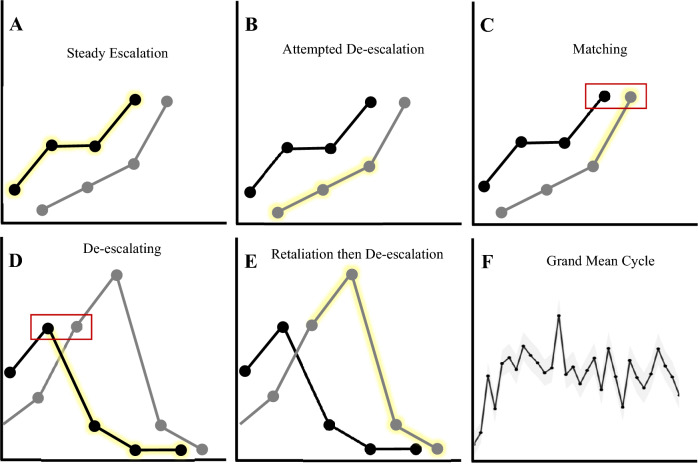
Oscillatory pattern of escalation and de-escalation. A prototypic pattern of aggressive behaviour in one couple, illustrating the contributions that prior level, velocity and acceleration make to the ongoing dynamics of conflict. (**A**) Each individual tends to match their opponent, but one person may initiate an increase in aggression; (**B**) Increases in aggression tend to lead to further increases, but the opponent may initially attempt to de-escalate by not retaliating; (**C**) If attempted de-escalation fails and aggression continues the opponent will then likely retaliate, matching the aggressor; (**D**) Increases in aggression from the opponent tend to lead to a reduction in aggression from the original aggressor, but the opponent may continue even after the original aggressor has de-escalated; (**E**) After the opponent has retaliated they may then de-escalate, resulting in a return to matching, and allowing the cycle to continue; (**F**) The cyclic nature of the conflict is consistent enough across dyads to be visible in a grand-mean plot representing the average blast level at each round.

This general model describes the prototypic conflict that occurs in our paradigm, a pattern that is illustrated in Fig. 4 (participants are both referred to here as ‘opponents’, because each member of the pair is both aggressor and target over time). If opponent one is escalating, they tend to continue (Fig. 4A); in response, opponent two may initially attempt to de-escalate (Fig. 4B); if opponent one continues to increase the rate of their escalation (i.e., they positively accelerate), opponent two has little option but to match them (Fig. 4C); leading to opponent one de-escalating (Fig. 4D); opponent two’s aggression then likely continues, but after retaliating, they de-escalate (Fig. 4E); and the cycle continues. As we discuss below, we call this cyclic pattern the "high beam effect" due to its similarity to the (unsafe) nighttime driving practice of mutual high beam blinding. In fact, this cycle is consistent enough across dyads to be visible in a grand-mean^57^ plot representing the average blast level selected by the winner of each round. Analysis reveals that the oscillation is statistically significant (according to Sinusoidal Curve Fitting via Nonlinear Least Squares^58^, cf. Figure 4F; amplitude: a = 0.16, *p* = 0.03; frequency: b = 0.87, *p* = 3e-15; phase shift: c = 2.96, *p* = 0.004; vertical shift: d = 3.42, p < 2e-16; residual standard error = 0.289; convergence tolerance = 4e-6).

**Fig. 5:**
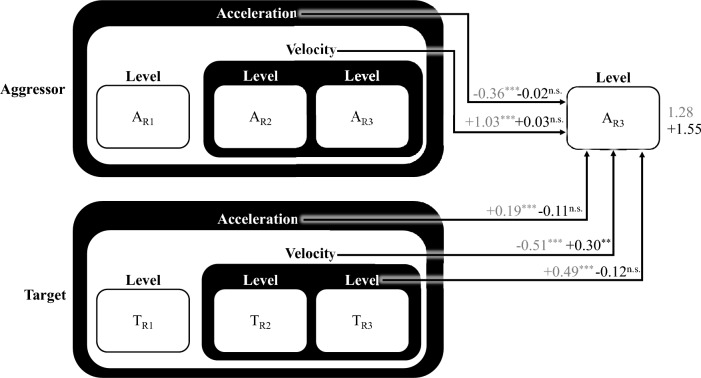
cross-lagged, coupled oscillatory model of women’s aggression. A model of aggression with only trials where the aggressor is a woman, allowing us to examine how women behave depending on whether the target is a man or another woman. The model can be read and interpreted as described in Fig. a3. In addition, all predictor paths (and the intercept) include two estimates. Values shown in grey represent women-women pairs, acting as the reference pair absorbed into the intercept. Values in black represent women-man pairs, representing the difference between woman-woman and woman-man pairs (i.e., how the predictors change, rather than an absolute value of the estimate for woman-man pairs). For example, when all predictors are zero and we have a woman-woman pair, the predicted blast level is 1.28. The predicted blast for a woman-man pair (all else being zero) is the base estimate (1.28) plus the difference for woman-man pairs (1.55), resulting in a blast of 2.83. The same addition rules hold for estimates of the influence of predictor variables. For example, a one unit increase in aggressor velocity predicts a 1.03 unit increase in blast level for woman-woman pairs (bringing the estimated blast to 2.31). For woman-man pairs, there is an additional predicted increase of 0.03, such that the estimated blast is 3.89 (i.e., 1.28 + 1.55 + 1.03 + 0.03). Significant predictors are indicated using ** for *p* < 0.01; *** for *p* < 0.001 and the model explains ~ 75% of variance in the aggressor’s next blast level selection.

With these variables and behaviour patterns in mind, we now consider women’s aggression towards different target genders (illustrated in Fig. 5). As in the general model, this woman-aggressor model explains ~ 75% of the variance in blast level selection. As expected, the target’s last blast level has a strong influence on the aggressor’s blast level selection (regardless of target gender; *p* =  < 0.001, B = 0.49, β = 0.50, bf = 177), and there was no significant difference in this predictor when the (woman) aggressor was paired with a man or another women. Further, the role of velocity and acceleration replicates that seen in the general model: the aggressor’s velocity increases subsequent aggression (*p* =  < 0.001, B = 1.03, β = 1.06, bf = 3e + 13) and their acceleration reduces subsequent aggression (p = 0.001, B = -0.36, β =  − 0.64, bf = 2e + 5); whereas the target’s velocity reduces subsequent aggression (*p* = 1e-11, B = -0.51, β =  − 0.52, bf = 20) and their acceleration increases aggression (*p* = 0.001, B = 0.19, β = 0.35, bf = 0.5; though this Bayes Factor is anecdotal, indicating that Bayesian versions of our model with and without this factor performed similarly). Critically, however, only one of these factors varied as a function of the gender of the target: when the aggressor’s opponent was a man, her tendency to de-escalate in response to her opponent’s escalation (velocity) was significantly reduced (*p* = 0.019, B = 0.30, β = 0.31, bf = 0.028; though the Bayes Factor for the target’s velocity suggests that it is unlikely to be robust across different model structures and thus we use caution in interpreting this particular finding). In total, therefore, the model supports the claim that women are more aggressive with men than other women (*p* = 0.001, B = 0.53, β = 0.62, bf = 3.64), but as noted above, the increase in aggression largely reflects a response to men initiating it. All model specifications, including a standardized version of the same model and a fully Bayesian equivalent, are provided in Appendix D.

### Is men’s aggression driven by risky impulsivity?

**Fig. 6:**
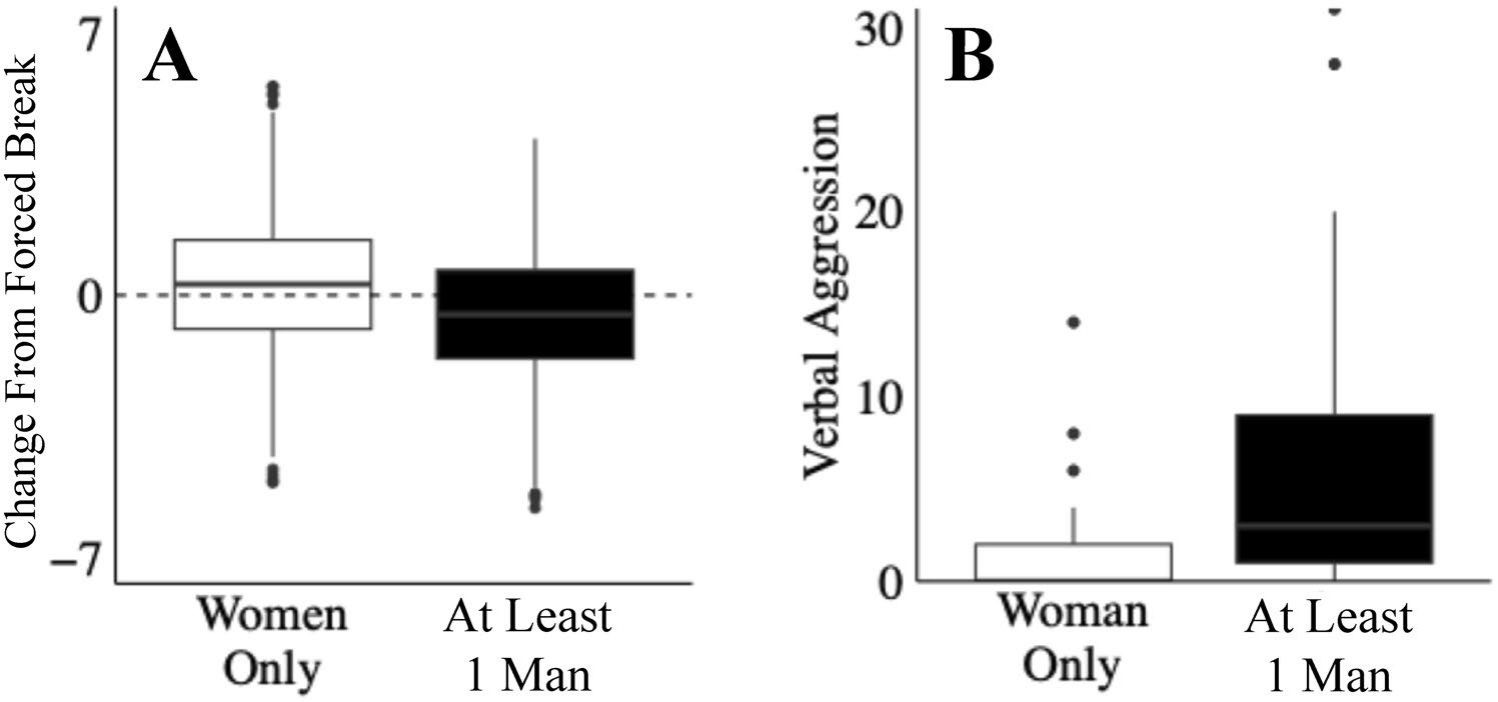
Men’s impulsivity or women’s non-physical aggression?. We predicted that men would be more impacted by our Forced Break condition due to their greater propensity to engage in impulsive behaviour. (**A**) As predicted, aggression in pairs containing at least one man was significantly decreased by forced breaks, and aggression in woman-woman pairs was not affected. Note that since our data was between participants, the subtraction visually represented in Panel A is a simulated subtraction built from our distributions (i.e., using bootstrapping), but all reported stats are from our raw data, not the simulated subtraction. (**B**) Given women’s reported aversion to specifically physical forms of aggression, we analysed verbal aggression from audio recordings of our task. We found that the patterns observed for physical aggression by aggressor-target pairing is identical for verbal aggression, where woman-woman pairs used significantly less aggression than pairs containing at least one man. Data represents counts of verbalizations.

The analysis and modelling outlined above shows that within our paradigm, reactive aggression is higher in dyadic interactions involving at least one man, and that this reflects a combination of men’s tendency to ‘ignite’ conflict, and women’s willingness to respond in kind when men exhibit aggression. While these data explain why aggression is lower when women interact with each other, they do not explain why men were so much more likely to initiate heightened aggression. To address this question, our experiments also included a manipulation that was designed to ‘check’ impulsive increases in aggression in a state of provocation (i.e., the manipulation prevents behaviour driven by negative urgency) via the introduction of forced breaks that required participants to pause before selecting a blast level (thus preventing impulsive aggression). Based on previous research that suggests men are more likely than women to act impulsively^1–3^, we anticipated that men should show a greater reduction in aggression during our Forced break Condition. In line with our prediction, analysis reveals that forced breaks significantly reduced aggression for pairs containing at least one man (*p* = 5e-11, r = 0.116, bf = 1e + 7), but had no effect for women-women pairs (*p* = 0.117, r = 0.040, bf = 0.073). Critically, the difference between these effects is significant (Fig. 6A; *p* = 9e-5, r = 0.314, bf = 402). In addition, the overall blast levels in women-women pairs are significantly above one (i.e., the lowest blast possible) in both the Immediate Response conditions (*p* = 2e-16, r = 0.76, bf = 7e + 106) and the Forced Break conditions (*p* = 2e-16, r = 0.77, bf = 3e + 80), indicating that the lack of a reduction in woman-woman pairs is not simply a floor effect. This analysis suggests, therefore, that men’s greater impulsivity contributes to their greater willingness to initiate aggression in the first instance.

### Post-hoc analysis: What about verbal aggression?

Given that we found women engaged in less physical aggression with other women than they did with men, we wondered if, instead, women were more engaged in non-physical forms of aggression with each other^1,9,59^. While our experiment was explicitly designed to measure physical aggression, audio recordings of the dyadic interactions allowed us to estimate verbal aggression during gameplay. We therefore used a machine learning model (Whisper^60^) to transcribe our participant’s speech throughout the task, and another pre-trained model (Empath^61^) to identify sentiments within our transcripts. We operationalized verbal aggression as the sum of sentiments relating to the pre-trained categories of hate, aggression, violence, dominant hierarchical, deception, fight, dominant personality, war, disgust, rage, anger, power, and competing. Due to the post-hoc nature of this analysis, speaker diarization (i.e., isolating each individual within a pair) was not possible, so scores were recorded for dyads and analysed as a function of gender pairing. Results matched our analysis of physical aggression, showing that verbal aggression was substantially lower in woman-woman pairs than all other pairs (Fig. 6B; *p* = 6e-4, r = 0.336, bf = 18.3; where verbal aggression data represents counts of aggressive verbalizations as specified by the Empath model^61^). These data suggest, therefore, that our women-women pairs were not simply substituting physical aggression with verbal aggression – they were less aggressive with each other overall.

## Discussion

We examined gender differences in aggression within a laboratory setting, using the Face-To-Face Competitive Reaction Time Task (FTF-CRTT)^36^. This experimental approach allowed us to measure reactive aggression during on-going dyadic interactions, tracking changes in aggressive behaviour over time. Our novel behavioural analysis and modelling showed that men were more aggressive than women, a gender difference that reflected three key factors: (i) elevated levels of aggression in men stemmed from an interaction between the gender of the aggressor and the gender of the target – men were equally aggressive with both genders, whereas women were less aggressive with other women; (ii) men were an order of magnitude more likely to initiate an increase in aggression than women, but despite initiating aggression less often than men, women were just as willing to respond aggressively when men started it; and (iii) men were more affected than women by the introduction of forced breaks that prevented participants from responding impulsively, providing evidence that gender differences in aggression are driven, at least to some extent, by men’s propensity for impulsive behaviour more broadly.

Claims regarding gender differences in aggression are controversial^1,62–65^, in part because differences observed in real-world behaviour vary with the form of aggression examined (e.g., physical vs. relational)^1,62–65^, cultural attitudes to gender equality^1,66–68^, and the gender of the target of aggression^8,20–22,32,64,69^. Research regarding women’s aggression suggests that (at least in cultures with high scores on indices of gender equality^66–68^) men and women in intimate relationships aggress against each other at roughly equal rates^8,32,63^ (though often with different consequences^20,21^). Survey evidence that women are as aggressive as men (at least by frequency) is hotly contested however, particularly by professionals working in domestic abuse services^8,32,63^. Here we provide clear experimental evidence that shows why gender differences in aggression are so complex in real world settings. Overall, our results revealed that men engaged in slightly more aggression than women in the lab, but analysis of the influence of both the aggressor and target demonstrated that this was due entirely to women-women pairs showing particularly low aggression in our study. Critically, when mixed-gender pairs were examined, women were just as aggressive as men. Thus, rather than reflecting only a simple, individual-level, gender difference in aggression, the overall difference in behaviour results from an interaction between the gender of the aggressor and target.

Our analysis did nonetheless reveal a clear gender difference: men were an order of magnitude more likely to *initiate* heightened aggression than women. In our task, women-women pairs often exhibited very little escalation, whereas in mixed-gender pairs, men typically initiated increased aggression and then (once this intention to use high aggression was evident) women retaliated by matching the higher level of aggression. We interpret this finding to suggest that when the likelihood of retaliation is uncertain (i.e., before each person has seen their opponent aggress, they don’t know whether their opponent will match them, escalate or de-escalate), women are more cautious than men and avoid using aggression, thereby avoiding aggressive retaliation from their opponent (a pattern that leads to the overall gender-pair disparity seen in the present study). This interpretation is strengthened by our post-hoc analysis of verbal aggression, which also revealed lower levels of aggression between women-women pairs than all other pairs and showed that woman-woman pairs were not simply using verbal aggression in place of physical aggression. Taken together, therefore, our data suggests that women’s lower overall levels of aggression reflects a reduced willingness to initiate heightened aggressive conflict (compared to men) – not a reduced willingness to behave aggressively per se.

Critically, our paradigm placed tight constraints on the frequency and severity of aggressive behaviour: participants were required to administer a sound blast on every round in which they won the reaction time game (i.e., a ‘zero’ response was not possible; though it is worth noting that 75db is unlikely to cause discomfort) and, for ethical reasons, the severity of aggression was limited to the maximum blast level allowed (110db). These task constraints meant that every participant had the same capacity to cause their opponent discomfort, unlike in real-world settings where men (on average) are more able to inflict physical harm on a woman than vice versa. Despite these constraints, the aggressive behaviour expressed by our participants was highly variable, and mean blast level selections were far from both floor and ceiling. As a result, during our conflict, participants could reasonably have inferred that more aggressive behaviour from themselves was likely to prompt higher degrees of aggressive retaliation from their partner. However, if men were still perceived as more physically intimidating than women despite the constraints of the lab setting, this is not evident from the patterns of aggression. Women participants were not reluctant to initiate heightened aggression towards men specifically: rather, they tended to refrain from initiating aggression irrespective of their opponent’s gender. Our data, therefore, provide support for ‘threshold’ models of aggression^70^ in which women (on average) require a greater degree of provocation than men before they will initiate aggression. Given wider differences in risk-taking^2,3^ and punishment sensitivity^2,71^ between men and women, we would still expect women to be more reluctant than men to use high levels of aggression in real world settings, when the risk of retaliation is unconstrained (i.e., there is an implicit potential threat to life and limb). Nonetheless, our findings add to the literature showing that sex differences in aggression vary as a function of provocation^72,73^ and highlight the need for further experimental research examining how women’s behaviour varies with the severity of aggression from an opponent^e.g.,^^11,40^.

In addition to examining mean levels of aggression, we also examined how aggressive behaviour changed over time. To do so, we introduced a coupled model that incorporated data from all rounds of the task, allowing us to predict an aggressor’s behaviour from the proximal (prior) behaviour of both themself and their target. Specifically, we examined the impact of (blast) level, velocity (change in level from one round to the next), and acceleration (change in velocity over successive rounds). Modelling revealed that an aggressor’s future behaviour was influenced by both their own and their target’s past behaviour. Aggressors responded to an opponent’s velocity (escalation) by decreasing their aggression, but they increased their aggression when an opponent showed signs of acceleration. Interestingly, the opposite pattern held for the influence of an aggressor’s own past behaviour on their future aggression. That is, once an aggressor’s behaviour was escalating (i.e., velocity), they were more likely to continue escalating on the next round. Most importantly, perhaps, an aggressor’s increasing escalation (i.e., acceleration) served to inhibit their future aggression and led to de-escalation – suggesting a potential mechanism that an individual could use to regulate their own aggressive behaviour (in line with Interpersonal Circumplex models of dyadic interactions^74–76^).

To characterise the dynamics of conflict and make it more intuitive we describe it as the “high beam effect”, due to its similarity to the (unsafe) night-time driving practice of mutual high beam blinding. Specifically, imagine driving down an unlit street at night as you are approached by a driver whose high beams are on, blinding you. You may flash your high beams to warn them and usually they turn their high beams off. However, if the other driver keeps their high beams on, you might retaliate by turning on your own high beams to blind them as well. Once you pass, you turn your high beams off, and the cycle continues with the next driver you encounter. Our modelling of conflict dynamics (and the high beam effect specifically) carries practical significance, potentially suggesting that interventions for dyadic conflict should target pairs during conflict troughs (after the initial high beam warning flash, before mutual blinding) to avoid re-escalating after a de-escalation. To further illustrate this point, we have produced a browser-based simulator that allows users to experience what it is like to engage with a (statistical summary of) our players behaviour (please note that it may take up to 60s to load the simulator: https://ftf-crtt-1.onrender.com). Similarly, as noted above, interventions aimed at reducing aggression could leverage people’s tendency to de-escalate in response to their own accelerating aggression. Encouragingly, and consistent with our previous demonstration^36^ that conflict between romantic couples does not escalate to maximum and then remain there, the fluctuating dynamics of conflict revealed here stand in stark contrast with the steady escalation of aggression predicted by the Violence Escalation Cycle^26^.

Modelling also provided additional insight into the nature of gender differences in aggression between men and women in our lab setting. We examined how previous behaviour influenced future aggression in women, when interacting with either men or women. As for the overall model, these data show that an opponent’s previous level of aggression has a strong positive influence on an aggressor’s future behaviour, but this effect was significantly reduced when women were in conflict with men. In addition, although women reduced their aggression in response to an opponent’s increasing aggression (i.e., escalation) when their opponent was female, this effect was significantly smaller when their opponent was male. That is, women were more willing to de-escalate in response to a woman’s show of aggression, than they were to a man’s equivalent show of aggression—although as noted in the results, Bayesian modelling suggests that the gender difference in response to escalation is unlikely to be robust across different model structures. Given the lack of wider evidence about the dynamics of interpersonal conflict, we cannot be certain whether this gender effect is specific to the controlled conditions present in our experimental task. The difference in women’s sensitivity to aggression from men and women seen here may, for example, vary depending on the nature of the relationship (e.g., between friends and strangers), or as a function of the severity of the aggression (e.g., interacting with women’s greater punishment sensitivity). Regardless, the present data suggests that modelling the dynamics of conflict over time can provide additional insight into the nature of gender differences in aggression.

The present studies and analysis represent an important methodological innovation, particularly in relation to our use of pairs of real participants, interacting in real-time, face-to-face. Critically, the patterns of aggression evoked by our FTF-CRTT paradigm were not random; participants changed their behaviour based on their opponent’s behaviour, and thus the unique dynamics shown here represent meaningful patterns of behaviour between two real people. Whilst our face-to-face version of the CRTT is new, previous studies have examined *virtual* interactions (participants were in different rooms) between pairs of participants^26,37^, revealing similar dynamic patterns of behaviour (most notably, the kind of cyclic escalation-de-escalation pattern presented here^37^). Notably, however, a study by Koch et al. (2024)^37^ using virtual dyadic interactions showed evidence that participants respond differently to real and fake opponents. In this case, the fake opponent delivered a pattern of steadily escalating aggression, regardless of the real participant’s behaviour (creating a different overall pattern of conflict between two real participants and a real participant vs. a fake opponent). By contrast the vast majority of previous studies using TAP/CRTT variants use only a single participant and a fake opponent, with the fake opponent typically “choosing” punishment levels randomly^25^. Given the findings presented here, it is our view that studies using this random selection approach with their fake opponents are severely limited in their interpretability, as they force participants to interact with random, meaningless patterns of behaviour from the fake opponent, even though we know that real participants respond dynamically to each other. Using a non-random approach like steady escalation is likely an improvement on purely random punishment selection, but fake opponents that do not respond dynamically (as real people do) are still inherently limited in their ecological validity.

Further, our use of a face-to-face design means that not only do participants know without a doubt that they are competing against a real person, but they can also see and verify that the sound blasts they select are sent to their opponent (and vice versa). Moreover, because we use face-to-face interactions our opponents can see the consequences of their aggressive behaviour, reflected in their opponent’s reactions. Put simply, therefore, we believe that the face-to-face CRTT offers a substantial increase in ecological validity compared to previous studies using two real participants competing virtually^26,37^. Consequently, the present article was able to provide novel insights into the complex temporal dynamics of conflict between dyads over time. These methodological improvements can also explain why we observed significant and meaningful gender differences where previous studies using virtual competition between real dyads did not^37^. We note, also, that the lack of gender-related findings in some TAP variation studies may sometimes be explained by their use of non-physical indices of aggression (e.g. deductions from a monetary reward). As discussed in the introduction, women are more willing to use non-physical aggression than physical aggression, so we might expect smaller (or non-existent) gender differences in studies using non-physical punishments^37^.

**Fig. 7:**
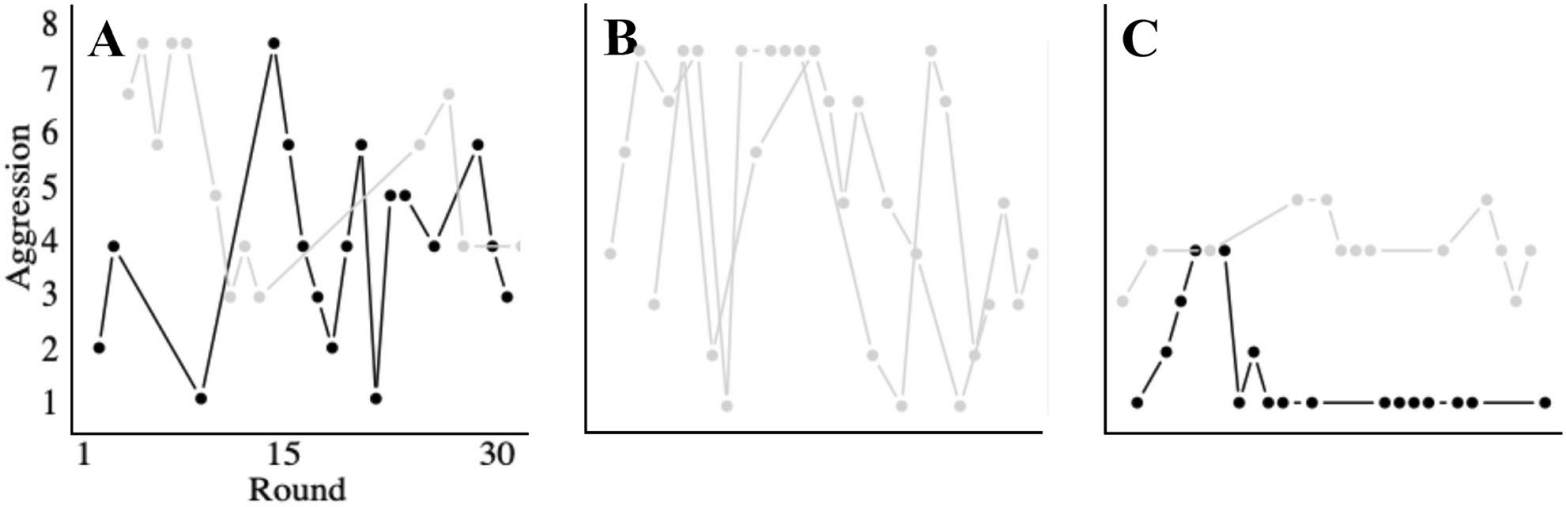
Breaking the mould. While we present evidence of both gender differences in aggression, and interactions between the gender of the aggressor and target, it is important to recognise that these patterns are averages across a large sample of data. In recognition of the variability within the data, and to avoid misleading claims about the average patterns being a complete description of behaviour, here we highlight patterns of behaviour that break the mould. Illustrative examples of real conflicts between pairs taking part in our studies show that: (**A**) Although aggression in mixed-gender pairs was predominantly initiated by men (black lines), not all aggression was initiated by men – sometimes women (grey lines) started it. (**B**) While most woman-woman pairs did not escalate above minimal aggression levels, some women used sustained, high levels of aggression. (**C**) While aggression was bi-directional and maintained by both individuals in most dyads, sometimes an individual sustained their own aggression in the absence of provocation (and in this case, it was the women in the pair, not the man, maintaining her own aggression).

Considering the sensitivity of the topic of gender differences in aggression, we wish to make our position regarding the limits of our findings explicit. Crucially, the analysis and models presented here are statistical summaries, based on individual response data from all participants, and therefore characterise behaviour at a group level (rather than representing individuals). Thus, although we show that men are an order of magnitude more likely to initiate heightened aggression than women, our findings should not be taken as an indication that men initiate *all* aggression. On the contrary, some of our female participants initiated aggression themselves and some of our male participants did not initiate aggression (as illustrated in Fig. 7A), although most of them did. In addition, while we show that women aggress less with other women than men, our findings should not be taken to suggest that women *never* use physical aggression against other women. While most women-only pairs used no more than minimal aggression against each other, some engaged in high levels of aggression (cf. Figure 7B). Equally, although our findings show that conflict is typically reciprocal, with aggression from both parties, they should not be taken to suggest that aggression is *always* fuelled by both people in a dyad. Indeed, we observed some dyads where one person sustained their own aggression without provocation from their opponent (Fig. 7C).

More broadly, our finding that women matched aggression from men should not be interpreted as evidence that women’s aggression is simply ‘self-defence’, or used to support victim-blaming (e.g., “if she just didn’t engage, he would have calmed down”). Defensive aggression specifically refers to aggression motivated primarily by fear and enacted when escape is impossible, whereas here participants were able to escape (e.g., by taking off their headphones or leaving the lab), and thus the concept of self-defence (as used by aggression researchers) does not apply. Equally, from a theoretical perspective aggression can only be considered defensive if it serves to protect oneself (or territory, resources, kin, etc.) from an attack^77–79^, which, again, does not apply to our task. Furthermore, although we have shown^36^ both partners’ negative emotion drives aggression during the FTF-CRTT, participants generally enjoy the task and the emotion expressed most often during it is actually happiness. We also highlight that the fact men initiate heightened aggression more often than women should not be interpreted as evidence that men are inherently aggressive ‘by nature’. On the contrary, our demonstration that the introduction of a brief forced break stopped men’s impulsive aggression in its tracks suggests that simple contextual changes can significantly alter behaviour. Finally, it is important to acknowledge that our participants were largely students at a university in a small UK town, almost all of whom reported their gender as either ‘man’ or ‘woman’, and although it lies beyond the scope of the current article, we note that the meaning associated with these gendered labels varies depending on many factors and is itself somewhat contested^80,81^.

The dyadic interactions studied here clearly differ from ‘real-world’ aggressive incidents in many ways, and it is important to acknowledge our study limitations, including that our primary data speaks only to physical forms of reactive^82,83^ aggression, as measured in a laboratory setting. Further, we attribute aggressive intent to the use of (especially louder) sound blasts in our sample, though it is possible that the motivations behind sound blast selection were not exclusively aggressive^e.g.,^^27^. We also did not test participants for their sensitivity to the sound used in the present study, so we cannot be sure that men and women experienced equivalent discomfort at each sound blast level. Nonetheless, our results complement wider survey-based research that also shows that women’s aggression appears to increase when the opponent is a man^11^. The greater temporal resolution of lab-based, as opposed to survey, measures allow us to suggest that this ‘target shift’ occurs because men are more likely than women to initiate an aggressive encounter, and that women interacting with men raise their aggression levels to match. This, in turn, might explain why reported rates of real-world aggression between non-intimate pairs (e.g., friends or strangers) are much higher among men than women^62,84^, while aggression from men and women in intimate pairs is often found to be roughly equal in frequency (though, of course, this varies depending on how aggression is measured, and the types of aggression examined)^8,32,63^. We recognise that other techniques capture aspects of real conflict that cannot be examined in the lab, and thus our findings need to be examined in real world contexts (where a wider array of factors may come into play). It is notable, however, that while the FTF-CRTT was not designed to examine verbal aggression (such that we could not isolate verbal interactions for each member of each pair), post-hoc analysis of verbal aggression recorded during the conflict nevertheless provided further support for our primary findings. Given that wider observational data shows women engage in mostly non-physical aggression^1^ during real-world conflict, further research is clearly required to better understand gender differences in, and the dynamics of, verbal aggression. Examining the interactions between aggressor-target gender with non-physical forms of aggression (e.g., verbal, relational, etc.) will, therefore, be an important topic for future research.

In sum, our results showed that men were more likely than women to initiate high levels of aggression in the lab, with the result that women-only dyads often expressed minimal aggression. This gender difference did not, however, reflect a total unwillingness among women to behave aggressively. Indeed, women matched their opponent’s physical aggression levels once these were raised, resulting in equal aggression from men and women in mixed-gender pairs. We were able to characterise these dynamic patterns of behaviour because of the dyadic and temporal nature of our FTF-CRTT paradigm, which measures aggressive behaviour between individuals over time, revealing nuances that are not commonly measured in aggression research. In addition, because we experimentally manipulated the opportunity for our participants to respond immediately, we were able to examine the mechanism that underpins these gender differences in aggression. Critically, we found that a forced break before responding reduced men’s aggression more than women’s, suggesting that higher levels of aggressive behaviour in men than women is driven, at least in part, by men’s greater propensity to act impulsively.

## Supplementary Information

Supplementary Information.

## Data Availability

De-identified data available at 10.5281/zenodo.17165808. Raw video/audio data is not available to protect participant anonymity. If you encounter any issues accessing the data, please contact the corresponding author (A.G.M.).

## Code availability

Data was analyzed in R and the analysis code is available along with the data at the DOI provided above. Custom code to run the experiment (and our browser-based player simulator) was written in Python and is available on GitHub (https://github.com/AnnahGrace01/FTF-CRTT), and archived with 10.5281/zenodo.12797202.
