# Supplementary material for: Men’s impulsivity underpins gender differences in aggressive behaviour
Source: Sci Rep. 2025 Oct 9;15:35215. doi: 10.1038/s41598-025-20114-6 (PMC12511576; doi:10.1038/s41598-025-20114-6)
Supplement: Supplementary file 1 — Supplementary Information. [file 41598_2025_20114_MOESM1_ESM.docx]

**Appendix**

***Appendix A.* Normality** ***Statistics***

******

***Note.*** “M” refers to men and “W” refers to women. When one letter is followed by another, the first letter represents the gender of the aggressor, and the second letter represents the gender of the target.

***Appendix B. General Models***

| **General Model Summary (all aggressor-target genders)** | | | |
| --- | --- | --- | --- |
|  | **Predicting Blast Level** | | |
| *Predictors* | *Estimates* | *CI* | *p* |
| (Intercept) | 2.00 | 1.62 – 2.38 | **<0.001** |
| velocity | 1.03 | 0.96 – 1.10 | **<0.001** |
| acceleration | -0.36 | -0.40 – -0.32 | **<0.001** |
| partner level | 0.44 | 0.37 – 0.51 | **<0.001** |
| partner velocity | -0.35 | -0.45 – -0.26 | **<0.001** |
| partner acceleration | 0.11 | 0.06 – 0.16 | **<0.001** |
| **Random Effects** | | | |
| σ^2^ | 1.25 | | |
| τ_00_ _id:pair_id_ | 1.01 | | |
| τ_00_ _pair_id_ | 0.11 | | |
| ICC | 0.47 | | |
| N _id_ | 60 | | |
| N _pair_id_ | 30 | | |
| Observations | 744 | | |
| Marginal R^2^ / Conditional R^2^ | 0.514 / 0.743 | | |

|  |  |
| --- | --- |

| **Standardized General Model Summary (all aggressor-target genders)** | | | |
| --- | --- | --- | --- |
|  | **Predicting Standardized Blast Level** | | |
| *Predictors* | *Estimates* | *CI* | *p* |
| (Intercept) | 0.08 | -0.05 – 0.22 | 0.216 |
| velocity | 1.06 | 0.99 – 1.13 | **<0.001** |
| acceleration | -0.62 | -0.70 – -0.55 | **<0.001** |
| partner level | 0.44 | 0.37 – 0.51 | **<0.001** |
| partner velocity | -0.36 | -0.46 – -0.26 | **<0.001** |
| partner acceleration | 0.20 | 0.12 – 0.29 | **<0.001** |
| **Random Effects** | | | |
| σ^2^ | 0.25 | | |
| τ_00_ _id:pair_id_ | 0.21 | | |
| τ_00_ _pair_id_ | 0.02 | | |
| ICC | 0.47 | | |
| N _id_ | 60 | | |
| N _pair_id_ | 30 | | |
| Observations | 744 | | |
| Marginal R^2^ / Conditional R^2^ | 0.514 / 0.743 | | |

| **Bayesian General Model Summary (all aggressor-target genders)** | | |
| --- | --- | --- |
|  | **Predicting Blast Level** | |
| *Predictors* | *Estimates* | *CI (95%)* |
| Intercept | 0.72 | 0.56 – 0.88 |
| velocity | 0.29 | 0.27 – 0.31 |
| acceleration | -0.10 | -0.11 – -0.09 |
| partner_level | 0.10 | 0.08 – 0.13 |
| partner_velocity | -0.09 | -0.12 – -0.06 |
| partner_acceleration | 0.03 | 0.01 – 0.04 |
| **Random Effects** |  |  |
| τ_00_ _id:pair_id_ | 0.27 | 0.08 – 0.44 |
| τ_00_ _pair_id_ | 0.33 | 0.24 – 0.44 |
| Observations | 744 | |
| R^2^ Bayes | 0.755 | |
| Shape (ϕ; Gamma Dispersion) | 8.69 | |

***Appendix C. Women Aggressor Models***

| **Women Aggressor Model Summary** | | |  |
| --- | --- | --- | --- |
|  | **Predicting Blast Level** | | |
| *Predictors* | *Estimates* | *CI* | *p* |
| (Intercept) | 1.28 | 0.74 – 1.82 | **<0.001** |
| pair specific [WM] | 1.55 | 0.66 – 2.44 | **0.001** |
| velocity | 1.03 | 0.90 – 1.17 | **<0.001** |
| acceleration | -0.36 | -0.45 – -0.28 | **<0.001** |
| partner level | 0.49 | 0.34 – 0.64 | **<0.001** |
| partner velocity | -0.51 | -0.71 – -0.30 | **<0.001** |
| partner acceleration | 0.19 | 0.09 – 0.29 | **<0.001** |
| pair specific [WM] × velocity | 0.03 | -0.15 – 0.22 | 0.728 |
| pair specific [WM] × acceleration | -0.02 | -0.13 – 0.09 | 0.693 |
| pair specific [WM] × partner level | -0.12 | -0.31 – 0.07 | 0.215 |
| pair specific [WM] × partner velocity | 0.30 | 0.05 – 0.55 | **0.019** |
| pair specific [WM] × partner acceleration | -0.11 | -0.24 – 0.02 | 0.085 |
| **Random Effects** | | | |
| σ^2^ | 1.20 | | |
| τ_00_ _id:pair_id_ | 0.89 | | |
| τ_00_ _pair_id_ | 0.00 | | |
| ICC | 0.43 | | |
| N _id_ | 42 | | |
| N _pair_id_ | 27 | | |
| Observations | 536 | | |
| Marginal R^2^ / Conditional R^2^ | 0.565 / 0.751 | | |

***Note.*** In this and all following models, WM represents instances where women aggress against men. The WW group is the reference group and is therefore absorbed into the intercept.

| **Standardized Women Aggressor Model Summary** | | | |
| --- | --- | --- | --- |
|  | **Predicting Standardized Blast Level** | | |
| *Predictors* | *Estimates* | *CI* | *p* |
| (Intercept) | -0.15 | -0.33 – 0.03 | 0.112 |
| pair specific [WM] | 0.59 | 0.27 – 0.91 | **<0.001** |
| velocity | 0.94 | 0.82 – 1.07 | **<0.001** |
| acceleration | -0.56 | -0.69 – -0.44 | **<0.001** |
| partner level | 0.50 | 0.35 – 0.66 | **<0.001** |
| partner velocity | -0.54 | -0.75 – -0.32 | **<0.001** |
| partner acceleration | 0.35 | 0.16 – 0.54 | **<0.001** |
| pair specific [WM] × velocity | 0.03 | -0.14 – 0.20 | 0.728 |
| pair specific [WM] × acceleration | -0.03 | -0.21 – 0.14 | 0.693 |
| pair specific [WM] × partner level | -0.12 | -0.31 – 0.07 | 0.215 |
| pair specific [WM] × partner velocity | 0.32 | 0.05 – 0.58 | **0.019** |
| pair specific [WM] × partner acceleration | -0.21 | -0.44 – 0.03 | 0.085 |
| **Random Effects** | | | |
| σ^2^ | 0.28 | | |
| τ_00_ _id:pair_id_ | 0.21 | | |
| τ_00_ _pair_id_ | 0.00 | | |
| ICC | 0.43 | | |
| N _id_ | 42 | | |
| N _pair_id_ | 27 | | |
| Observations | 536 | | |
| Marginal R^2^ / Conditional R^2^ | 0.565 / 0.751 | | |

| **Bayesian Women Aggressor Model Summary** | | |
| --- | --- | --- |
|  | **Predicting Blast Level** | |
| *Predictors* | *Estimates* | *CI (95%)* |
| Intercept | 0.39 | 0.18 – 0.62 |
| pair gender [WM] | 0.62 | 0.27 – 0.96 |
| velocity | 0.33 | 0.28 – 0.37 |
| acceleration | -0.11 | -0.13 – -0.08 |
| partner level | 0.14 | 0.09 – 0.19 |
| partner velocity | -0.14 | -0.21 – -0.08 |
| partner acceleration | 0.05 | 0.02 – 0.08 |
| pair gender [WM] × velocity | -0.04 | -0.10 – 0.02 |
| pair gender [WM] × acceleration | 0.00 | -0.03 – 0.04 |
| pair gender [WM] × partner level | -0.05 | -0.11 – 0.01 |
| pair gender [WM] × partner velocity | 0.08 | 0.00 – 0.16 |
| pair gender [WM] × partner acceleration | -0.03 | -0.07 – 0.01 |
| **Random Effects** |  |  |
| τ_00_ _id:pair_id_ | 0.36 | 0.26 – 0.47 |
| τ_00_ _pair_id_ | 0.14 | 0.01 – 0.34 |
| Observations | 536 | |
| R^2^ Bayes | 0.746 | |
| Shape (ϕ; Gamma Dispersion) | 8.73 | |
|  |  | |

***Appendix D. Impact of Sample and Gender***

| **Gender Only Model** | | | | |
| --- | --- | --- | --- | --- |
|  | **Gender Only** | | | |
| *Predictors* | *Estimates* | *std. Error* | *CI* | *p* |
| (Intercept) | 2.57 | 0.19 | 2.20 – 2.94 | **<0.001** |
| pair gender [WM] | 1.30 | 0.26 | 0.78 – 1.83 | **<0.001** |
| pair gender [WM] | 1.23 | 0.27 | 0.71 – 1.76 | **<0.001** |
| pair gender [WM] | 1.82 | 0.51 | 0.82 – 2.81 | **<0.001** |
| Observations | 158 | | | |
| R^2^ / R^2^ adjusted | 0.184 / 0.168 | | | |

| **Sample Only Model** | | | | |
| --- | --- | --- | --- | --- |
|  | **Sample Only** | | | |
| *Predictors* | *Estimates* | *std. Error* | *CI* | *p* |
| (Intercept) | 3.83 | 0.14 | 3.56 – 4.10 | **<0.001** |
| sample [Friends] | -1.02 | 0.23 | -1.47 – -0.57 | **<0.001** |
| Observations | 158 | | | |
| R^2^ / R^2^ adjusted | 0.115 / 0.109 | | | |

***Note.*** These comparisons of the relative impact of the sample (friends vs. couples) and gender are provided for completeness. The reference category is the couples sample and is thus absorbed into the intercept.

| **Sample + Gender Model** | | | | |
| --- | --- | --- | --- | --- |
|  | **Sample + Gender** | | | |
| *Predictors* | *Estimates* | *std. Error* | *CI* | *p* |
| (Intercept) | 3.17 | 0.32 | 2.54 – 3.80 | **<0.001** |
| sample [Friends] | -0.75 | 0.33 | -1.39 – -0.10 | **0.023** |
| pair gender [WM] | 0.78 | 0.35 | 0.09 – 1.46 | **0.027** |
| pair gender [WM] | 0.69 | 0.35 | -0.00 – 1.39 | 0.051 |
| pair gender [WM] | 1.96 | 0.50 | 0.97 – 2.96 | **<0.001** |
| Observations | 158 | | | |
| R^2^ / R^2^ adjusted | 0.211 / 0.191 | | | |

***Note.*** Each Bayes Factor represents the relative evidence for one model over the other given the data. Here, a large BF suggests greater evidence for the first model in a comparison than the second. For example, the BF of ~56 suggests that there is 56 times more evidence in favour of the model with sample and gender than the model with only sample. In all these models, we used Jeffreys-Zellner-Siow (JZS) priors, a minimally informative prior selected due to our lack of preconceptions about the influence of gender vs. dyad type on aggressive behaviour. Overall, this analysis suggests that gender has a meaningful impact in our data, and the differences we observe are unlikely to be the result of just the different samples between our first and second included study.
